# Arsenic and Microorganisms: Genes, Molecular Mechanisms, and Recent Advances in Microbial Arsenic Bioremediation

**DOI:** 10.3390/microorganisms12010074

**Published:** 2023-12-30

**Authors:** Vladimir U. William, Hilbert D. Magpantay

**Affiliations:** Department of Chemistry, De La Salle University, 2401 Taft Avenue, Manila 0922, Philippines; vladimir_william@dlsu.edu.ph

**Keywords:** arsenic, heavy metal, microbial bioremediation, tolerance genes

## Abstract

Throughout history, cases of arsenic poisoning have been reported worldwide, and the highly toxic effects of arsenic to humans, plants, and animals are well documented. Continued anthropogenic activities related to arsenic contamination in soil and water, as well as its persistency and lethality, have allowed arsenic to remain a pollutant of high interest and concern. Constant scrutiny has eventually resulted in new and better techniques to mitigate it. Among these, microbial remediation has emerged as one of the most important due to its reliability, safety, and sustainability. Over the years, numerous microorganisms have been successfully shown to remove arsenic from various environmental matrices. This review provides an overview of the interactions between microorganisms and arsenic, the different mechanisms utilized by microorganisms to detoxify arsenic, as well as current trends in the field of microbial-based bioremediation of arsenic. While the potential of microbial bioremediation of arsenic is notable, further studies focusing on the field-scale applicability of this technology is warranted.

## 1. Introduction

Arsenic (chemical symbol: As; atomic number: 33; atomic mass: 74.921 amu) is a steel-grey metalloid that is naturally present in the environment but is most commonly found in rocks and soils, particularly in sulfide minerals [1,2]. While arsenic can exist in four oxidation states (As(III), As(V), As(0), As^−3^), the most common are the trivalent arsenite, As(III), and the pentavalent arsenate, As(V) [3]. Ecological niches differ in the form of arsenic present in them—As(III) prefers anoxic and reducing conditions, while aerobic environments have a preponderance of As(V) [4,5]. However, between the two oxyanions, As(III) is considered more toxic due to its high mobility in both aqueous and solid phases [6].

Currently, the World Health Organization estimates that at least 140 million people worldwide are exposed to arsenic at levels above provisional guideline values [7]. Considering the classification of arsenic as a Group 1 carcinogen and its declaration as the most pervasive hazardous substance found in the environment, its continuous, widespread impact has renewed recent interest in it as a pollutant of concern and has pushed the United States Centers for Disease Control and Prevention to put it at the top of the ATSDR’s Substance Priority List [8,9]. The impact of arsenic contamination is particularly seen in vulnerable regions highly reliant on groundwater as a primary source of potable water [10,11,12]. Global notoriety of arsenic was reached when an estimated 125 million people in Bangladesh were exposed to inorganic arsenic from contaminated tube wells, constituting what the World Health Organization calls “the largest mass poisoning of a population in history” [13]. In addition, the incessant use of soil amendments [14], as well as the constant conversion of land for agricultural and urban use [15], has led to a substantial increase in arsenic in areas with normally low concentrations.

One technique consistently gaining traction in dealing with arsenic contamination is bioremediation, or the use of suitable living microorganisms to convert environmental pollutants to an innocuous state [16]. Bioremediation is known for its relative ease of application [17], environmental sustainability [18], and cost-effectiveness [19]. In the case of arsenic bioremediation, it has been observed that microorganisms usually achieve bioremediation by reducing arsenic mobility through biotransformation of arsenic to its various ionic species. 

**Figure 1:**
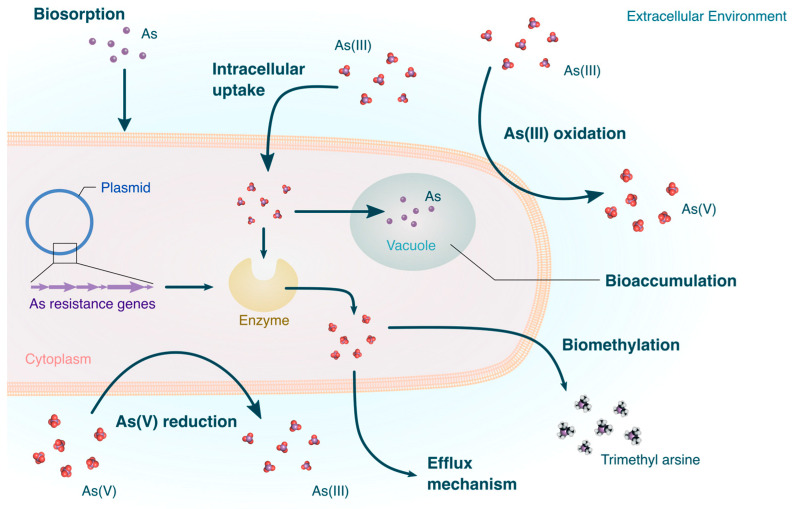
Overview of some of the mechanisms used by microorganisms to tolerate environmental arsenic.

To provide a broader overview of the current understanding of microbial arsenic detoxification, the general framework for this review begins with a description of the gene systems found in microorganisms known to be activated in response to arsenic stress. This is followed by the enzymes upregulated by the previously discussed gene systems and the complementary biochemical processes involved to treat arsenic. Figure 1 provides a general schematic of some of the mechanisms discussed in this review Then finally, a summary of the most recent, representative microorganisms used in arsenic bioremediation are provided, as well as some of the trends, challenges, and future approaches the field must face. 

## 2. Methods

A review of the available literature on arsenic bioremediation was conducted mainly on four electronic databases, namely, MDPI, PubMed, ScienceDirect, and Google Scholar. Search terms included the following free text words: “arsenic”, “arsenic bioremediation”, “arsenic biomethylation”, “microbial methylation of arsenic”, “biological volatilization of arsenic”, and “microbial arsenic resistance”. Other relevant literature was also found through backward search of already found articles as well as new articles that referenced the original paper. 

Inclusion criteria for the journal articles were the article was written in the English language, the study utilized microorganisms to treat arsenic, the article provided insights into the genes and biochemical mechanisms involved in microbial arsenic detoxification, and the study explored the use of microorganisms in combination with other methods to treat arsenic. An additional inclusion criterion used for Section 5, “Recently Isolated Arsenic Tolerant Microorganisms and Approaches Used in the Microbial Bioremediation of Arsenic”, was that the study must have been published within the last 10 years. Studies on combined bioremediation/physicochemical approaches were excluded if the focus was on the bioreactor design.

## 3. The Genetic Basis of Microbial Arsenic Detoxification

Due to the ubiquitous nature of arsenic in the environment, it is generally assumed that evolution has conferred all living things the necessary molecular machinery to detoxify arsenic [20,21]. As mentioned, the two most naturally abundant forms of arsenic are As(III) and As(V). Between the two, however, As(III) is more biologically important due to its ability to disrupt protein function by strongly binding to sulfhydryl groups, reducing cysteines, and effectively preventing proper protein folding. While As(V) also exerts toxic cell function by abrogating phosphate anion transporters, the danger lies in its susceptibility to be reduced to the more toxic As(III) [22]. Selective pressure from constant environmental arsenic stress have allowed microorganisms to evolve niche-specific gene expressions to facilitate arsenic uptake [23]. This includes the genes which confer the ability to utilize As(III) as an electron donor and As(V) as an electron acceptor [24]. 

### 3.1. The aio Gene Systems

Presumed habitat vestiges of primordial life, such as thermal vents, were found to contain arsenic in the form of As(III), leading researchers to conclude that primitive microorganisms may have had bioenergetic use for these ions [25,26,27]. Since survival is reliant on the immediate detoxification of the more toxic As(III), it has been surmised that the mechanisms of As(III) oxidation is the most ancient As detoxification system, which recent phylogenetic analyses have confirmed [28]. In various prokaryotes and Archaea, the most prevalent As(III) oxidation system is governed by the *aio* (also known as *aso*, *aox*, or *aro*) gene cluster [29,30]. 

The archetypal *aio* system—the *aioBA* operon—was first identified and completely sequenced from the β-proteobacteria *Herminiimonas arsenicoxydans*. It is a bicomponent, regulatory pair composed of *aioB* and *aioA*, which respectively encodes for the small, Rieske 2Fe-2S cluster and the large molybdopterin subunit of the enzyme, arsenite oxidase (Aio) [31]. Structural elucidation of the Aio expressed by *Alcaligenes faecalis* provided a more intimate look at not only the enzyme, but also what came to be known as “arsenic gene islands”. These gene islands are functionally related As(III)-resistance genes in mutually independent microorganisms [32,33].

**Figure 2:**
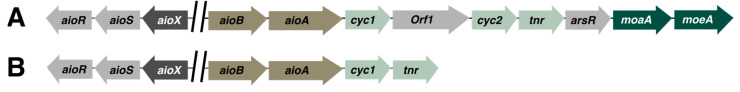
Examples of genetic organization of aio operons in a few arsenic-resistant microorganisms. Arrows represent open reading frame and the direction of transcription. Orthologs are represented in the same color. (**A**) *Thiomonas arsenitoxydans* [38], (**B**) *Acidovorax* sp. NO-1 [39].

Explorations into the *aio* gene islands of various microorganisms revealed that differences in the organization of intergenic sequences between flanking genes, as well as homologous *aioBA* systems, are unique to each organism (see Figure 2). An in silico investigation of gene clusters of 55 phylogenetically distinct bacterial species confirmed this by concluding that although the *aioBA* domain is widespread across As(III) oxidizing species, multiplicity in the occurrence, orientation, and sequence of specific regulatory genes is only present in some species, as is seen in the case of the *aioXSR* operon, which can only be found in *Proteobacteria* [34]. Interestingly, it was previously thought that the *aioXSR* system—composed of *aioX*, which encodes for a periplasmic-binding protein (PBP); *aioS*, a sensor kinase; and *aioR*, a response regulator—was highly conserved and ubiquitous in all As(III) oxidizing microorganisms because of its ability to tightly regulate the expression of *aioBA* [35,36,37]. Genetic synteny between As(III) resistance genes and genes which are activated under phosphate-induced stress (such as *pho*, *pst*, and *phn*) was also established leading to the conclusion that co-expression of both these genetic domains may confer additional resistance to high concentrations of arsenic [34].

Another example of the divergence of As(III)-resistance genes can be found in the acidophilic *Thiomonas arsenitoxydans*, which was found to cotranscribe two unique genes, *orf1* and *cyc2*, together with the *aioBA* cluster [38]. *orf1*, which encodes for a DNA binding metalloregulator, and *cyc2*, which encodes for a monohemic cytochrome c, suggests that these distinct genes regulate both the oxidation of As(III), as well as the activation of the operon in the bacterium. Another study also highlighted the novelty of the *T. arsenitoxydans aio* operon from other arsenite oxidizers by showing that in the presence of As(III), the bacterium induces *aioF* together with the classical *aioBA* operon, acting as the main regulator of the *aio* cluster. 

Other important homologs of the *aio* system include the *aioE* of *Agrobacterium tumafeciens* GW4 [40]. *aioE* encodes for the oxidoreductase, AioE, which is not only essential for arsenic resistance but also plays a role in the generation of NADH in the electron transport chain. Intriguingly, *aioE* is found in the same operon as the As(V) reductase gene, *arsC*, which could mean that regulation and expression of each gene may be affected by the other. It is important to note, however, that during in-frame deletion of either *aioE* or *arsC*, only the As(III) oxidation pathway is primarily affected, which may explain why the *aioE* is the dominant phenotype observed in *A. tumafeciens* GW4.

### 3.2. The ars Gene Systems

Since the archaic Earth contained much higher concentrations of environmental arsenic, life would have had needed to evolve arsenic-resistance genes to thrive [20]. Additionally, the eventual oxygenation of the Earth’s atmosphere lead to the conversion of inorganic arsenic into As(V), giving microorganisms selective pressure to evolve genes required to reduce As(V) [41]. These environmental challenges have allowed microorganisms to diversify their genetic toolkit and gave rise to arsenic-resistance genes, encoded in the *ars* operons [21].

**Figure 3:**
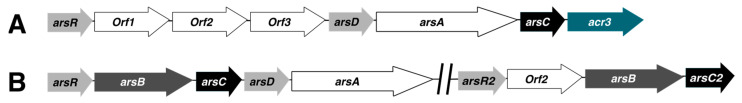
Examples of genetic organization of ars operons in a few arsenic-resistant microorganisms. Arrows represent open reading frame and the direction of transcription. Orthologs are represented in the same color. (**A**) *Bacteroides vulgatus* [42], (**B**) *Bacillus* sp. [43].

Serendipitously, the *ars* genes were isolated not from directly examining arsenic-resistant microorganisms but from the study of antibiotic-resistance genes in *Staphylococcus aureus* more than 50 years ago. The isolated plasmid, pI258, has been found to not only confer resistance to antibiotics but also to other metals, arsenic included. Using similar techniques, the R773 plasmid was isolated from *E. coli*, from which the main arsenic resistance phenotype is derived: the *arsRDABC* operon (see Figure 3). A few Gram-positive bacteria, meanwhile, possess *arsRBC*, a three-component gene system homologous to *arsRDABC*. Over the years, numerous studies focused on the isolation of arsenic-resistance genes followed suit, allowing the identification of more *ars* and *ars*-like genes.

Each individual component of the *arsRDABC* gene cluster codes for a specific type of protein related to arsenic removal: The *arsR* encodes for a trans-acting transcriptional repressor member of the SmtB/ArsR family [44]; *arsA* codes for an ATPase [45], which together with the ArsB efflux pump can form their own ATP-dependent As(III) efflux pump [46]; *arsD* codes for a another trans-acting regulator metallochaperone, which has the ability to bind As(III) and to expel it via transfer to the ArsAB efflux pump [47,48]; and *arsC,* which codes for the canonical prokaryotic As(V) reductase, ArsC [49]. In fungi, the equivalent As(V) reductase is coded by the *ACR2* gene [50]. In addition to the common *ars* genes, some microorganisms, such as the obligate anaerobe *Bacteroides vulgatus*, contain the *acr3* gene, which codes for an inorganic arsenic efflux pump [42].

### 3.3. The arr Gene Systems

In contrast with the other gene systems, the genes that code for dissimilatory As(V) reduction (also known as respiratory As(V) reduction) are a later evolutionary response to environmental arsenic stress. Known as the arr gene cluster, to date, only Bacteria and Archaea have been shown to express arr. Because of this, research on the arr genes and its resultant proteins is still in its nascent stages.

First described in *Shewanella* sp. ANA-3, the arr genes code for an As(V) reductase, similar to the ars cluster. The arr operon codes for the heterodimer respiratory As(V) reductase ArrAB, made up of a large and small subunit encoded by arrA and arrB, respectively. The large ArrA subunit is composed of a bismolybderopterin guanine dinucleaotide cofactor and an [4FE-4S], both of which act as the binding and catalytic site of As(V). Meanwhile, the small ArrB subunit contains four [4Fe-4S] clusters [51,52].

While continued searches for microorganisms which can express genes related to arrA are underway, phylogenetic analysis suggests that the eventual prevalence of As(V) in the environment coincided with the split of Bacteria and Archaea, explaining the specificity and preponderance of arr genes in only the two domains [53].

## 4. Biomolecular Pathways Involved in the Microbial Detoxification of Arsenic Related to Bioremediation

### 4.1. Divergent Arsenic Detoxification Strategies Used by Microorganisms

In the biogeochemical cycling of arsenic, microorganisms play a central role in the transformation of arsenic from one form to another. Depending on the ecological niche, the predominant arsenic species may vary, which is why genes for arsenic resistance are encoded by all microorganisms. The process of biotransformation ultimately governs the speciation and mobilization of arsenic in all environmental matrices. Because of this, the most well-known mechanisms of arsenic detoxification in microorganisms involve the metabolic use of arsenic as an electron donor or acceptor in respiratory redox processes. The two processes are distinct in how they metabolically utilize environmental arsenic. When microorganisms are faced with As(III), cellular energy is gained from the oxidation of the As(III) by using it as an electron donor. In contrast, As(V) is reduced by using it as the terminal electron acceptor in anaerobic cellular respiration. Microorganisms can also transform arsenic into various organic compounds which have important implications in arsenic toxicity. 

### 4.2. Microbial As(III) Oxidation

The oxidation of the more toxic As(III) is the prime arsenic detoxification mechanism used by microorganisms. Recent phylogenetic analyses have conclusively proven that the enzymes required for As(III) oxidation predate all other arsenic detoxification processes [54]. While this important process was first observed as early as 1918 [55], only recently with the development of new, more sophisticated structural elucidation techniques have researchers been given a clearer picture of the regulatory mechanisms and proteins involved in the process. Because of the rise in interest in exploiting microbial As(III) oxidation mechanisms for potentially novel bioremediation approaches, various papers have also reviewed the topic [30,56].

**Figure 4:**
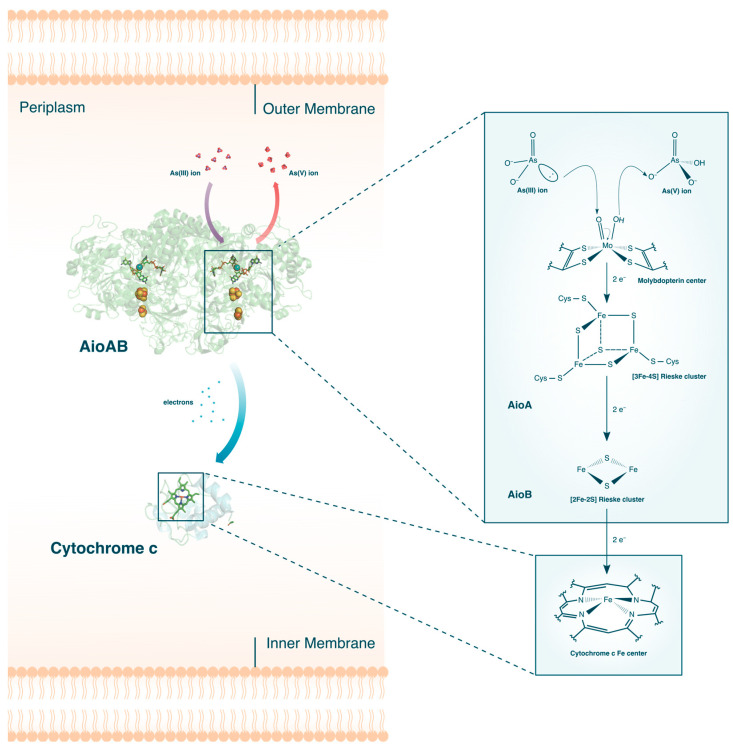
The aerobic oxidation of As(III) in bacteria. The crystal structures for AioAB [57] and Cytochrome c [59] are shown. Description of the electron flow in the active sites are given in the text.

The aerobic oxidation of As(III) is primarily catalyzed by the As(III) oxidase, Aio (or AioBA) (see Figure 4). When challenged with As(III) stress, upregulation of aioBA genes occurs, which codes for the two subcomponents of Aio: the large α-subunit, AioA, and the small β-subunit, AioB. Crystal structures [32,57] of the protein complex revealed that the two subunits can be further subdivided into their individual redox active sites. The first active site is a molybdenum (Mo)-centered bis-pterin guanine dinucleotide surrounded by a network of protein residues and water molecules all connected by hydrogen bonds and salt bridges. Also found inside the AioA domain is a [3Fe-4S] Rieske cluster, while AioB contains a [2Fe-2S] Rieske cluster. Once expressed, these proteins are then translocated either to the periplasm or the cytoplasm and coupled with a c-type cytochrome [58]. 

A combination of stopped-flow spectroscopy and isothermal titration calorimetry revealed that the Aio catalyzed oxidation of As(III) into As(V) occurs via four distinct electron transfer events [60]. When As(III) enter the vicinity of the funnel-liked opening of AioA, highly polar residues attached to the molybdopterin molecule binds the As(III). An electron pair from the bound As(III) performs a nucleophilic attack on the Mo(VI) center of the molybdopterin molecule, effectively reducing Mo(V) to Mo(IV). The reduction of Mo(V) and simultaneous oxidation of As(III) to As(V) happens almost instantaneously, with a reported rate of >4000 s^−1^. This is followed by sequential electron transfers from the molybdenum center to the two Rieske clusters, where the Fe and S atoms are also reduced rapidly. The last step, and coincidentally the rate-limiting step, is the electron transfer from the [2Fe-2S] Rieske cluster in AioB to the final electron acceptor, cytochrome c.

Interestingly, comparison of different As(III)-oxidizing microorganisms revealed that some species not only possess unique ligands surrounding the redox active sites but also differ in which cellular membranes Aio translocates to [61]. For instance, the Aio of Alcaligenes faecalis, whose structure was the first to be completely determined, contains a canonical disulfide bridge anchoring the AioB Rieske cluster [32]. Previously thought to be essential to the catalytic activity of the Rieske proteins, the importance of the disulfide bridge was put into question after the more recent structural elucidation of the native Aio of *Pseudorhizobium banfieldiae* sp. NT-26 (previously *Rhizobium* sp. NT-26 [62]), which found it completely devoid of the disulfide bridge [57]. By comparing the native Aio of *Ralstonia* sp. 22, which has a disulfide bridge surrounding its Rieske proteins, with a mutant incapable of forming a disulfide bridge in its AioB, it was confirmed that no loss of function happens when the disulfide bridge is removed. Whether Aio attaches to the periplasm or cytoplasm appears to be also variable as the Aio of *Ralstonia* sp. 22 prefers the cytoplasmic membrane, while the Aio of *Rhizobium* sp. NT-26 translocates to the periplasmic membrane. The full depth of the variation in As(III) oxidases in microorganisms remains enigmatic.

### 4.3. Microbial As(V) Reduction

Microorganisms can reduce As(V) through two different mechanisms: the first is through the activation of cytoplasmic As(V) reductases via the ars operons and the second is through dissimilatory As(V) reduction encoded in the arr gene system. 

**Figure 5:**
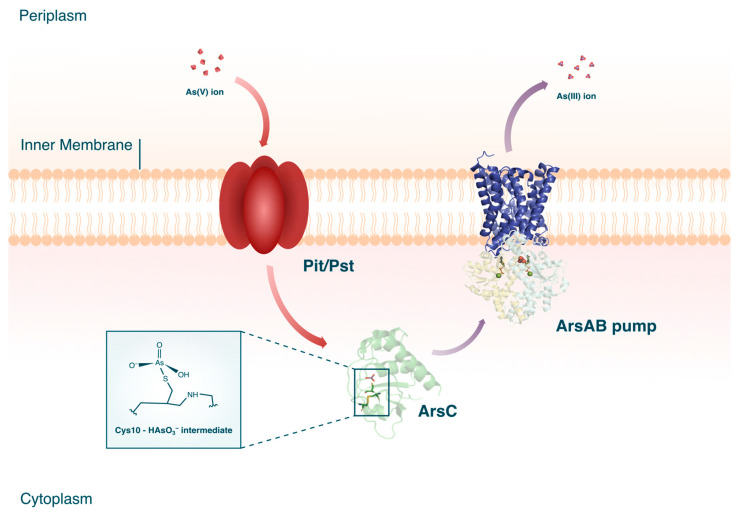
Cytoplasmic As(V) reduction in microorganisms. The crystal structures for ArsC [64], ArsA [65], and ArsB (PDB accession code AF_AFP45946F1) are shown. The crystal structures for ArsA and ArsB are combined in the figure to form the ArsAB efflux pump.

Cytoplasmic As(V) reductases are believed to be ubiquitous among microorganisms. Current knowledge on the ars genes separates the ArsC proteins into three different clades (see Section 3.2) which, while evolutionarily divergent, all effect the same molecular function [63]. The biochemical cascade of cytoplasmic As(V) reduction begins with the intracellular uptake of As(V) through phosphate membrane systems (see Figure 5). Due to As(V) being a chemical analog of phosphorus, As(V) ions can enter the bacterial cell through the periplasmic binding proteins, phosphate inorganic transport (Pit), or phosphate-specific transport (Pst). Once inside the cytoplasmic milieu, arsC coding for the As(V) reductase, ArsC, is upregulated. The conventional ArsC from *S. aureus* plasmid pI258 is a monomeric phosphatase spanning 135 amino acids with three bound cysteine residues [64]. These cysteine residues are essential for the enzymatic activity of ArsC as these facilitate the catalytic reduction of As(V) through a series of redox reactions: first, the thiol of the first cysteine (Cys^10^) covalently binds As(V) and reduces it to As(III); the resulting intermediate (Cys^10^-HAsO_3_^−^) is then attacked by the thiol of the second cysteine (Cys^82^), concurrently releasing the As(III); and the last step is the regeneration of the ArsC through the reduction of the intermediate by the third cysteine (Cys^89^).

In the case of the *E. coli* plasmid R773 ArsC [66], the two reducing cysteines are provided each from glutathione (GSH) and glutaredoxin (Grx). In contrast to the S. aurerus pI258 ArsC, once As(V) enters the vicinity of the *E. coli* ArsC, three arginine residues form a charged pocket around the single catalytic cysteine (Cys^12^) residue, becoming the active site where the As(V) is bound [67]. Insight into the crystal structure of ArsC have concluded that the reaction between Cys^12^ results in the subtle change in the geometry of the enzyme with it first forming a pentavalent complex with the As but eventually collapsing to a thioarsenate adduct, ArsC (Cys^12^-As) ([68]. The ArsC (Cys^12^-As) forms an intermediate with the GSH cysteine, while the Grx cysteine reduces As(V) to As(III) as well as cleaves the disulfide bond of the intermediate, effectively releasing the As(III) and restoring the ArsC. The resulting As(III) is then transported and expelled outside the cytoplasm via the ArsAB efflux pump. Compared to prokaryotes, up to now, only one arsenate reductase has been identified in eukaryotes: Acr2p. 

### 4.4. Microbial Arsenic Biomethylation

Biomethylation, also sometimes known as biovolatilization, is defined as the biological conversion of metals and metalloids to both volataile and nonvolatile methylated metabolites [69]. First observed in fungi, biomethylation plays an important role in microbial arsenic detoxification as well as in the biogeocycling of arsenic. The ability of microorganisms to both biomethylate and resist arsenic is conferred through the activation of the arsM gene [70]. Comparing orthologs of the arsM gene in different microorganisms revealed its prokaryotic origin, explaining its similarity to the ars operon genes used by bacteria [71].

To date, the most plausible biochemical pathway for arsenic biomethylation is still the one proposed by Challenger et al. [72,73]. For methylation of arsenic to proceed, reduction of As(V) to As(III) is required, followed by two more enzyme-catalyzed reduction reactions. For each reduction reaction, concomitant methylation of each As(III) intermediate occurs, with the final product being trimethylarsine. Later studies have confirmed S-adenosylmethionine (SAM) to be the previously elusive methyl group donor. 

## 5. Recently Isolated Arsenic Tolerant Microorganisms and Approaches Used in the Microbial Bioremediation of Arsenic

### 5.1. Bacteria

Numerous bacterial species are known to not only tolerate high concentrations of arsenic but also transform it from one ionic species to another. Since the ionic species of arsenic decides its toxicity as well as its mobility in the environment, bioremediation approaches usually rely on the biotransformation capability of microorganisms. Table 1 gives a summary of some of the recently isolated arsenic-resistant bacteria and the presumed mechanisms they use to tolerate both As(III) and As(V).

The oxidation of As(III) is of particular interest to bioremediation studies due to its potential application in large scale pre-treatment of arsenic-contaminated groundwater. In typical water treatment processes, As(V) can be easily removed through conventional physico-chemical techniques such as adsorption or ion exchange [81]. However, treating As(III) is considered more challenging due to its solubility and its low affinity for absorbents [82]. To remedy this, most operations rely on converting As(III) to As(V) through chemical pre-oxidation, which, by itself, is inherently inefficient due to the slow reaction rate and its tendency to form potentially toxic oxidation byproducts. Recent technologies are now shifting towards utilizing the inherent ability of some microorganisms to oxidize As(III) and coupling it with other arsenic removal strategies. One example used As(III) oxidizing bacteria belonging to the class *Alphaproteobacteria* and *Betaproteobacteria* and integrated it with a continuous-flow electrocoagulation system to remove arsenic in simulated tap water to below the 10 µg/L As(III) limit set by the World Health Organization [83].

Some of the most effective microorganisms used in arsenic bioremediation, both in batch reactor experiments and in situ applications, are sulfate-reducing bacteria (SRB) [84]. Investigating the effect of inoculating *Desulfovibrio vulgaris* in waters to simulate arsenic-contaminated aquifers, researchers were able to observe the successful reduction of As(V) with and without sulfate amendment [85]. To probe the biotransformation of arsenic compounds, researchers screened for native arsenic-tolerant microorganisms in dam tailings from a gold mine from Iran [86]. Out of thirty-seven microbial isolates, *Alishewanella agri* was found to tolerate up to 140 mM of As(III) and 600 mM As(V). Under bioreactor conditions, the bacterium was also shown to reduce up to 94.7% of As(V) and oxidize 8% of the As(III). A similar study aimed at isolating bacteria from soils around a gold mine in Brazil was able to identify a hyper-tolerant strain of *Bacillus* cereus as well as *Lysinibacillus boronitolerans* resistant to up to 3000 ppm of As(III) which can bioaccumulate 69.38%–71.88% As(III) and 82.39%-85.72% As(V) [79]. 

By using locally isolated, sulfate-reducing bacterial consortia, researchers were able to completely remove arsenic content from acid mine drainage from a mine in Carnoulès, France [87]. Analysis of the microbial community of the consortium revealed the presence of known sulfate reducers *Propionibacteraceae* sp. and *Desulforsporosinus* sp. SRB has also been shown to transform arsenic even at low pH [88]. In the treatment of arsenic-contaminated acid mine drainage, locally isolated SRB were shown to tolerate and remove up to 80% arsenic under a pH of 3.5. 

Bacterial species belonging to the genus *Bacillus* are among the most robust microbes used in the treatment of arsenic in soils. In a study which screened for potential arsenic hyper-tolerant microorganisms from a gold mine in Brazil, a strain of *Bacillus cereus* was isolated and found to be resistant to up to 3000 ppm of As(III) in lab conditions [79]. Taking advantage of the arsenic resistance of the bacterial strain, its ability to bioaccumulate and oxidize As(III) in vitro was also assessed. Although a 94.9% efficiency in reducing As(III) was reported, the mass balance of the percentage of As(III) and As(V) bioaccumulated within cell walls did not correspond to the concentrations of the ions in the culture medium. Speciation of the As(V) ions was speculated to account for the disparity, and a follow-up study to prove this is planned. Nevertheless, a successful reduction in arsenic to concentrations fitting global standards was successfully shown.

Perhaps one of the most remarkably arsenic-tolerant bacterial strains isolated so far is a strain of *Bacillus firmus* isolated from soil near the Lonar lake in India [78]. The strain, characterized as *Bacillus firmus* L-148, showed exceptional tolerance to exceedingly high concentrations of As, capable of thriving in concentrations of up to 247,241 ppm of As(III) and 299,686 ppm of As(V). The hyper-tolerant strain was also able to oxidize As(III) in the presence of other heavy metals and in alkaline conditions. While the latter is expected due to the fact that the lake where it is indigenous is naturally basic, the bacteria were capable of oxidizing As(III) even in buffered conditions. To date, no other bacterial species has shown tolerance to such high concentrations of arsenic.

Other notable bacterial species that can biotransform arsenic are *Corynebacterium* [89], *Pseudomonas* [77], *Micrococcus* [76], *Roseomonas,* and *Nocardiodes* [75]. Tolerated arsenic concentrations of these bacterial species in bioreactor experiments varied from 150 ppm to 2585 ppm for As(III) and 375 ppm to 29,968 ppm for As(V). 

### 5.2. Fungi

Compared to bacteria, fungi have the advantage of being deemed as the dominant living biomass present in soils [90]. The intimate association between fungi and soil is due to the low degree of shear strain experienced by soils, allowing the development of the fungal hyphal network [91]. Despite this, the potential of fungi in bioremediating arsenic in soils remains restricted. Nevertheless, recent studies have gradually started exploiting the various metabolic capabilities of these organisms against arsenic contamination. Table 2 gives a summary of some of the recent fungal candidates for arsenic bioremediation.

Explorations into filamentous fungi such as Penicillum [92,95], Fusarium [93], Trichoderma [94], Humicola [96], and Aspergillus [93,95] showed notable resistance to high arsenic concentrations. Screening of As(V)-contaminated agricultural soil in India revealed that a strain of Penicillium coffeae can tolerate As(V) concentrations of up to 37,461 ppm in vitro [92]. The fungus was also able to tolerate the same concentration of As(V) under basic conditions, whether living, dead, or as treated biomass. However, the exact mechanism the fungus utilizes in tolerating As(V) remains unknown. 

Among the numerous filamentous fungi used for arsenic bioremediation, the most used and studied is *Aspergillus*. Past research [97,98,99] has already attested to the efficacy of using indigenous *Aspergillus* sp. present in soils to mitigate arsenic contamination. Recently used *Aspergillus* systems were able to resist as much as 10,000 ppm As(V) [93] and 3000 ppm As(III) [95].

Rather than relying on redox reactions, the more prevalent route used by fungi to detoxify inorganic arsenic is through biomethylation [95,98,100]. During fungal biomethylation, arsenic is transformed into both volatile methylated compounds, namely, monomethylarsine, dimethylarsine, and trimethylarsine, and nonvolatile compounds such as methylarsonate and dimethylarsonate [69]. Fungal species such as *Aspergillus*, *Trichoderma*, and *Penicillium* are presumed to activate these genes in response to arsenic stress.

There is still diverging consensus on whether arsenic biomethylation can be classified as a detoxification process. While some consider arsenic biomethylation as a means to abate the toxic effects as it reduces the affinity and solubility of the ions in environmental substrates [101], recent interpretations have questioned the claim of reduced toxicity of arsenic in alkylated form. Using molecular models in human cell lines, research has shown that monomethylarsonic acid and dimethylarsinic acid, two metabolites of arsenic biomethylation, induced structural perturbations in cell membranes [102]. This puts into question the merits of using fungi over bacteria in potential large-scale arsenic bioremediation experiments.

### 5.3. Microbial Consortium

A relatively unexplored approach to the bioremediation of arsenic is the identification and application of arsenic-resistant mixed microbial consortia. When using pure cultures, bioremediation efficiency may be limited by the difficulty of maintaining pure cultures, especially in in situ applications. Another important consideration is the ability of pure cultures to bioremediate complex contamination scenarios. Efficient bioremediation using mixed microbial cultures has consistently been observed for a myriad of pollutants, which has been attributed to the symbiotic and co-metabolic action between the different species in a specific consortium [103].

A recent study aimed at identifying the microbial community composition of a household sand filter used for arsenic detoxification of groundwater revealed that the consortium is dominated by manganese-oxidizing bacteria as well as other nitrifying microorganisms. The authors conclude that the predominance of these specific microorganism may be attributed to the abundance of the presence of reduced forms of arsenic, iron, and nitrite ions [104]. In another study, pure and mixed cultures of *Acidithiobacillus* spp. were used in the bioleaching of arsenic and other heavy metals from mine tailings from a copper/silver mine. When compared in terms of bioleaching efficiency in ex situ experiments, the mixed cultures showed significant advantage over pure cultures [105]. 

Immobilization of As(III) under aerobic conditions was also achieved using thermoacidophilic consortium. By using granular activated carbon as a catalyst, a mixed culture comprised of mostly species from the genera *Sulfobacillus* and *Sulfolabales*, were able to remove as much as 0.9% *w*/*v* of As(III) in batch reactor experiments [106]. Despite obvious advantages in bioremediation experiments, there is still a dearth of studies focusing on the use of mixed microbial cultures for arsenic detoxification.

## 6. Challenges and Future Perspectives in the Microbial Bioremediation of Arsenic

While the abundance of microorganisms in various arsenic-contaminated environmental niches lends itself to unlimited, untapped potential for novel microbial strains, the isolation of most of these microorganisms, however, is often a great challenge. Despite numerous advances in bioinformatics, the vast majority of potentially exploitable microorganisms have yet to be identified. By using novel approaches, or sometimes, integrating various complex analytical techniques with so-called multi-omics approaches, recent research has been making great strides in overcoming this barrier.

By piecing together multi-omics analysis data of cultures derived from anoxic soils, researchers were able to determine the elusive identity of a bacteria capable of not only methylating arsenic in anaerobic conditions but also to actively detoxify the product [107]. Named *Paraclostridium* sp. EML, the bacteria were confirmed to express the *arsM* gene, as well as the genes *arsH*, *arsI*, *arsP*, and *arsR4*, which are all involved in the detoxification of methylated arsenic.

Another main challenge in using microorganisms for arsenic bioremediation is the transition from successful lab-scale microbial arsenic detoxification to field-scale, actively utilized applications. Microbial arsenic tolerance in in vitro experiments may not necessarily translate well when scaled-up to more practical levels, nor be more sustainable than physico-chemical remediation techniques. The reliance on indigenous microorganisms may also be limited by their innate metabolic rate to detoxify the contaminant.

To circumvent these limitations, studies focusing on large-scale arsenic bioremediation usually couple arsenic tolerant microorganisms with adsorption removal technologies to augment the removal efficiency of both processes. One study took advantage of the ability of SRB to drive the formation of biogenic pyrite for the removal of arsenic in groundwater [108]. SRBs, such as *Desulfovibirio desulfuricans*, can biomineralize pyrite directly, depending on the iron source readily available in the environment [109]. The growth of SRBs was first stimulated through the injection of a solution (consisting of organic carbon, ferrous iron, and sulfate) in specific sites in groundwater near an arsenic-contaminated aquifer. After 6 months, removal rates of up to 90% of total arsenic from the upgradient ground water were reported. Removal of the heavy metal is achieved though the adsorption and co-precipitation of the dissolved arsenic with the reduced sulfide during biogenic pyrite formation. 

Another study relied on the growth of native As(III) oxidizing microorganisms grown on a substrate of sintered glass and coarse sand to create a biofilter [110]. To test the efficiency of the biofilter at the household-scale, groundwater spiked with 100 µg L^−1^ of arsenic was allowed to circulate through the biofilter. Maximum oxidation efficiency was observed at a flow rate of 140 mL min^−1^ corresponding to a daily production of 400 L of drinking water. Interestingly, while the microbial community of the two substrates did not significantly differ, it was found that the coarse sand biofilters were more efficient in oxidizing As(III), reaching a maximum efficiency of up to 85%. Future studies may build upon the coarse sand biofilter and further couple it with an As(V) reduction setup to produce a mixed system design. 

## 7. Conclusions

While arsenic is a natural and inevitable part of the biogeochemical cycle, the rise in anthropogenic activities has led to its continued increase in arsenic concentrations in various environmental matrices. High arsenic concentration is considered a threat due to its recalcitrant nature as well as its capacity for highly toxic effects in plants, animals, and humans. Among all domains of life, microorganisms have been dealing with arsenic since life arose and are the most resilient to its lethal effects. Strides in elucidating the biochemical pathways of their ability to detoxify arsenic has allowed us to utilize their potential in bioremediation processes. However, there is still a dearth of knowledge on how we can tap into the full potential of arsenic-resistant microorganisms and scale-up existing strategies. With the rise of advanced biotechnological techniques, insights into the intimate association between microorganisms and arsenic may provide insight into how we can overcome its limitations. 

## Figures and Tables

**Table 1:** Arsenic-tolerant bacterial species.

Bacteria	Dominant Mechanism of Arsenic Tolerance	Maximum Tolerated Arsenic Concentration		Reference
		As(III)	As(V)	
*Brevibacillus* sp. KUMAs2	Plasmid-mediated	17 mM	265 mM	[74]
*Roseomonas* sp. L-159a	*aioA*, *aioB*	2 mM	50 mM	[75]
*Nocardioides* sp. L-37a	Activation of ArsC	5 mM	100 mM	
*Micrococcus* sp. KUMAs15	Upregulation of *aioB*	733 µM	400 mM	[76]
*Pseudomonas* AK1	Expression of the *aio* operon	13 mM	-	[77]
*Pseudomonas* AK9		15 mM		
*Bacillus firmus* L-148	Expression of *ars* and *aio* operon	75 mM	-	[78]
*Bacillus cereus*	As(III) efflux	24 mM	-	[79]
*Lysinibacillus boronitolerans*				
*Brevibacterium* sp. CS2	*aioB*	40 mM	275 mM	[80]

**Table 2:** Arsenic-tolerant fungal species.

Fungi	Dominant Mechanism of Arsenic Tolerance	Maximum Tolerated Arsenic Concentration		Reference
		As(III)	As(V)	
*Penicillium coffeae*	Unknown	-	500 mM	[92]
*Aspergillus oryzae* FNBR_L35	Biosorption, Biomethylation	-	71 mM ^a^	[93]
*Fusarium* sp. FNBR_B7, FNBR_LK5, FNBR_B3				
*Aspergillus nidulans* FNBR_LK1				
*Rhizomucor variqbilis* sp. FNBR_B9				
*Emericella* sp. FNBR_BA5				
*Trichoderma* sp. MG	Biomineralization	6 mM ^b^		[94]
*Aspergillus* sp. LMC 330.04	Biomethylation	0.0244 mM	0.0225 mM	[95]
*Penicillium* sp. LMC 331.03				
*Humicola* sp. 2WS1	*arsM*	38 mM	12 mM	[96]

^a^ Actual bioaccumulated arsenic ranged from 0.023 to 0.259 g kg^−1^; biovolatilized arsenic ranged from 0.23 to 6.4 mg kg^−1^. ^b^ ionic species unspecified.

## Data Availability

Data are contained within the article.
